# Physical Activity as Moderator of the Association Between *APOE* and Cognitive Decline in Older Adults: Results from Three Longitudinal Cohort Studies

**DOI:** 10.1093/gerona/glaa054

**Published:** 2020-02-28

**Authors:** Najada Stringa, Natasja M van Schoor, Yuri Milaneschi, M Arfan Ikram, Vieri Del Panta, Chantal M Koolhaas, Trudy Voortman, Stefania Bandinelli, Frank J Wolters, Martijn Huisman

**Affiliations:** 1 Department of Epidemiology and Biostatistics, Amsterdam Public Health Research Institute, Amsterdam UMC—Vrije Universiteit, the Netherlands; 2 Department of Psychiatry, Amsterdam Public Health Research Institute, Amsterdam UMC—Vrije Universiteit, the Netherlands; 3 GGZ inGeest, Amsterdam, the Netherlands; 4 Department of Epidemiology, Erasmus University Medical Center, Rotterdam, the Netherlands; 5 Laboratory of Clinical Epidemiology, InCHIANTI Study Group, LHTC Local Health Tuscany Center, Florence, Italy; 6 Department of Sociology, VU University, Amsterdam, the Netherlands

**Keywords:** Gene–environment interaction, Longitudinal Aging Study Amsterdam, InCHIANTI, Rotterdam Study

## Abstract

**Background:**

Previous studies have suggested that the association between *APOE* ɛ _4_ and dementia is moderated by physical activity (PA), but the results remain inconclusive and longitudinal data on cognitive decline are missing. In this study, we examine whether there is a gene–environment interaction between *APOE* and PA on cognitive decline in older adults using 9-year follow-up data of three cohort studies.

**Methods:**

We followed 7,176 participants from three longitudinal cohort studies: Longitudinal Aging Study Amsterdam (LASA), InCHIANTI, and Rotterdam Study for 9 years. PA was assessed with self-reported questionnaires and was categorized in low, moderate, and high PA. Cognitive function was assessed with the Mini-Mental State Examination (MMSE) and cognitive decline was defined as a decrease of three points or more on the MMSE during 3 years follow-up. We fitted logistic regression models using generalized estimating equations adjusting for age, sex, education, depressive symptoms, and number of chronic disease. Interaction between *APOE* and PA was tested on multiplicative and additive scale.

**Results:**

Cohorts were similar in most aspects but InCHIANTI participants were on average older and had lower education. *APOE* ɛ _4_ carriers had higher odds of cognitive decline (odds ratio [OR] = 1.46, 95% confidence interval [CI]: 1.29–1.64) while PA was not significantly associated with cognitive decline overall (moderate PA: OR = 0.87, 0.67–1.13; high PA: OR = 0.71, 0.36–1.40). There was no evidence for an interaction effect between PA and *APOE* ɛ _4_ in cognitive decline in older adults (*APOE* × moderate PA: *p* = .83; *APOE* × high PA: *p* = .90).

**Conclusions:**

Previous claims of a gene–environment interaction between *APOE* ɛ _4_ and PA in cognitive decline are not supported by our results.

Dementia is an increasing public health problem due to the increased life expectancy and the aging population (1,2). It has a complex genetic architecture and is largely influenced by lifestyle factors. In addition to dementia, there is an increasing burden of cognitive decline in the population.

Apolipoprotein E ε _4_ (*APOE* ε _4_) is the main genetic risk factor for dementia (3–5). Furthermore, the ε _4_ isoform of the lipoprotein is directly involved in the biological pathway of dementia and cognitive decline. It reduces the clearance of soluble amyloid beta (Aβ) from the brain and increases its deposition in senile plaques (6,7). In addition, several studies have shown that *APOE* ε _4_ carriers have more Aβ deposition in senile plaques in the brain compared to noncarriers (8,9).

Along with genetic factors, different lifestyle factors affect dementia and cognitive function in older individuals. Previous studies suggest that physical activity (PA) (10–12) has a positive effect on cognitive function. The exact mechanisms remain unknown but several theories are proposed such as enhancing cerebral perfusion, effects of PA on angiogenesis, neurogenesis, reduction of neuroinflammation, and reduction of Aβ deposition (13,14).

In the last years, a gene–environment interaction (GxE) between *APOE* and PA in Alzheimer’s disease (AD) has been suggested (15,16). A recent review by Bos and colleagues emphasizes the importance of identifying modifiable lifestyle factors for high-risk *APOE* ε _4_ carriers (17). The underlying biological mechanisms of this interaction are not completely understood but it is hypothesized that *APOE* ɛ _4_ carriers are more vulnerable to nonfavorable lifestyle factors compared to noncarriers. The study of Head and colleagues (14) showed that PA moderates the effect of *APOE* genotype on Aβ deposition with extra benefit in *APOE* ɛ _4_ carriers.

However, current literature on *APOE* ε _4_ by PA interaction is inconclusive and mainly focused on AD. Some studies suggest a stronger protective effect of PA on dementia in *APOE* ε _4_ carriers compared to noncarriers (18,19). Conversely, findings from Cardiovascular Health Cognition Study and Canadian Study of Health and Aging analyzing the effect of PA on dementia suggest a stronger protective effect of PA in *APOE* ε _4_ noncarriers compared to carriers (20,21).

Here, we aimed to explore whether PA might moderate the effect of *APOE* genotype on cognitive decline. Thus, we tested whether the GxE interaction between *APOE* genotype and PA was associated with cognitive decline in the general population of older individuals, using data from three prospective population-based cohort studies with longitudinal measurements.

## Methods

### Study Participants

This study includes data from three ongoing prospective cohort studies: the Longitudinal Aging Study Amsterdam (LASA), Invecchiare in Chianti (InCHIANTI), and Rotterdam Study (RS). A description of each study can be found in the Supplementary Material. The main analysis includes participants of Caucasian ethnicity, aged 55 years and older who underwent repeated cognitive assessment with the Mini-Mental State Examination (MMSE), and had a baseline MMSE score ≥18. Participants who were bedridden or in a wheelchair were excluded from the analysis. In total, 7,176 participants with 18,489 observations during 9-year follow-up were analyzed; respectively 1,736 participants with 4,255 observations from LASA, 843 participants with 2,132 observations from InCHIANTI study, and 4,597 participants with 12,102 observations from RS (Supplementary Figure 1). Mean follow-up period between two consecutive measurements was 3 years for LASA and InCHIANTI and 4 years for RS (Supplementary Table 1). Informed consent was obtained from all participants in each study and all studies have been approved by the respective Medical Ethics Committees.

### Assessment of *APOE*

In LASA, both phenotyping and genotyping of *APOE* were available. Phenotyping was performed at the Immunochemistry Laboratory of VUmc, Amsterdam (22). Genotyping was performed using the Axiom-NL array from Affymetrix (Avera Institute for Human Genetics, South Dakota) or the Infinium Global Screening Array from Illumina (Genetic Laboratory, Erasmus MC, Rotterdam, the Netherlands). *APOE* could be inferred from two single-nucleotide polymorphisms (SNPs), rs429358 and rs7412. A comparison between phenotyping and genotyping status of *APOE* showed 96.9% agreement between the two methods. Because more participants had phenotyping data (*N* = 2,233) compared to genotyping data (*N* = 1,021), the status determined by phenotyping was included in the analysis.

In InCHIANTI, *APOE* status was inferred from rs429358 and rs7412 SNPs genotyped on Illumina Infinium HumanHap550 chip at the Laboratory of Neurogenetics of the U.S. National institute on Aging.

In RS, *APOE* status was assessed at study entry using polymerase chain reaction on coded DNA samples.

### Assessment of PA

PA was assessed using different questionnaires per cohort which are described below. To make the PA measurements comparable between cohorts, PA was coded in three categories: low, moderate, and high PA (reference: low PA) following a similar procedure as described by Jonkman and colleagues (23).

In LASA, physical activity was assessed by using the validated LASA Physical Activity Questionnaire (LAPAQ) (24), based on the questionnaires by Vorrips and colleagues (25) and Caspersen and colleagues (26). The frequency and duration of the following activities in the previous 2 weeks was assessed: walking, cycling, light household activities, heavy household activities, and two most frequent sports. To take into account the intensity of each activity, metabolic equivalent of tasks (MET) was used. The MET-value is based on the ratio of work metabolic rate to resting metabolic rate and 1 MET is defined as 1 kcal/kg/h. The number of MET-hour for an individual spent on a specific activity was calculated by multiplying the MET-value by time spent on that specific activity (in hours) per week.

In InCHIANTI, PA was assessed using a modified standard interview-administered questionnaire (27) in which the participants provided data on current PA (28). Participants were asked about the frequency and duration of sports and recreational PA. By combining the responses and taking into account the intensity of each type of activity, participants were classified in one of the three categories:

(a) inactive, including participants who were completely inactive and those who performed low intensity PA.
(b) light PA, including participants who performed light intensity PA for 2–4 hours per week.
(c) moderate–high PA, including participants who performed at least moderate PA >3 hours per week or more and those who performed intense exercise many times per week.

In RS, PA was assessed by an adapted version of the validated Zutphen Physical Activity Questionnaire (26). The original questionnaire contains questions on walking, cycling, gardening, diverse sports, and hobbies. To obtain a more complete overview of physical activity, questions on housekeeping activities were added. Participants were asked how many hours they spent per week on these activities during the past 2 weeks. For some activities, like sports and gardening, participants were asked whether this activity was practiced only during summer or winter. When answered positive, the given period of time for that activity was divided by two (29). MET scores were assigned to each activity and subsequently the total MET hours/wk was calculated per each participant.

Study-specific tertiles were created based on the total MET hours/wk in LASA and RS.

### Assessment of Cognitive Decline

All three studies assessed cognitive function at each visit using MMSE, a 20-item scale ranging from 0 to 30 with higher scores indicating better cognitive function (30). A decrease of three or more points in MMSE in the following visit was considered as cognitive decline. The same definition for cognitive decline has been used in other cohort studies (31,32). Furthermore, results from the LEILA study, using the reliable change indices method with repeated measurements every 1.5 years, found that a mean MMSE decline of three or more points in 3 years is considered a reliable change in population-based cohorts of older adults (33).

### Assessment of Other Cognitive Tests in LASA

More specific aspects of cognition such as memory and information processing that are compromised in dementia were assessed in LASA. Episodic memory was assessed using the 15 Words Test, the Dutch version of the Auditory Verbal Learning Test (34). The test consists of 15 words that have to be learned during 3 trials. After every trial the respondent is asked to recall as many words as possible. After a distraction period of 20 minutes, the respondent is asked to name again the words he learned. The total number of words learned during three tests is the recall score (range 0–45). The number of words reproduced after 20 minutes is the delayed recall score (range 1–15) (22).

Information processing speed was assessed using an adjusted version of the Alphabet Coding Task-15 and has been described by Piccinin and Rabbit (35). The test was done in three cycles of one minute and the mean score of the three trials is used in the analysis (22).

These tests were either not available or different from the ones used in LASA in the other cohort studies; therefore, the analyses were only performed in LASA participants.

### Assessment of Covariates

The following covariates were considered: age (years), sex, education (less than 9 years of education, 9–12 years of education, more than 12 years of education), clinically relevant depressive symptoms (Center for Epidemiological Studies Depression Scale ≥16), and number of chronic disease (0–1, 2, >2 diseases).

A detailed assessment of covariates is described in the Supplementary Material.

Supplementary Table 1 presents an overview of the number of participants per wave, methods, and instruments used to assess exposure, outcome, and covariates in each cohort.

### Statistical Analysis

The main effect of APOE ɛ _4_ and PA and then their interaction effect (*APOE* × PA) on cognitive decline were assessed using generalized estimating equations (GEE) with exchangeable correlation structure, taking into account repeated measurements. A binary logistic model was used to assess the unadjusted effect estimates. In Model 1, we adjusted for sex (dichotomous), age at baseline (continuous), and education (categorical, reference category: less than 9 years of education). Subsequently, we adjusted for clinically relevant depressive symptoms (dichotomous) and presence of chronic disease (categorical, reference category: 0–1 chronic disease) in Model 2.

After running GEE in each study site, the results were meta-analyzed using random effects to take into account possible heterogeneity between studies. The I^2^ statistic was calculated, which measures the percentage of variation between studies that is not due to chance: I^2^ < 25% is considered low heterogeneity. To determine whether the heterogeneity was statistically significant, the chi-square test of heterogeneity was used (36). For analyses indicating a high degree of heterogeneity, we performed three leave-one-out meta-analyses removing one cohort per time, in order to check the impact of potential outlier data on the results.

### Sensitivity Analysis

Several sensitivity analyses were performed to test the robustness of the results against different aspects of cognitive function and scales of PA. First, since the reported levels of PA can be influenced by cognitive function, a sensitivity analysis was done including only individuals with good cognitive function at baseline (MMSE ≥24). Furthermore, to avoid loss of information due to the categorization of PA, a sensitivity analysis was conducted with PA assessed as continuous measure (total MET hours/wk divided by standard deviation) for studies in which continuous measures were available (ie, LASA and RS). Because interaction on the additive scale may reflect biological interaction better than interaction on the multiplicative scale (37) the relative excess risk due to interaction (RERI) was calculated according to the procedure proposed by Knol and colleagues (38), with 95% confidence intervals calculated by bootstrapping (*N* = 1,000).

### Extra Analyses in LASA

Analyses assessing different aspects of cognition as memory (total recall score, delayed recall score) and information processing speed that might be more sensitive than MMSE were done in LASA participants. There are no clear cutoffs for what can be considered decline in the 15-word test and coding task; therefore, we used as outcome the difference in scores between two measurements. Linear regression models using GEE with exchangeable correlation structure was fitted.

Analyses in LASA and RS were done using IBM SPSS Statistics version 21.0 (IBM Corp, New York) and in InCHIANTI using STATA/SE 12.0. RERI was calculated using R (version 3.5.1). The meta-analysis of the results was done using the rmeta package and bootstrapping was done using boot package in R. The figures were made using Review Manager version 5.3.5 a tool from the Cochrane Collaboration (39).

## Results

A total of 7,176 individuals were included in the main analysis. The sample characteristics per study are shown in Table 1 and the baseline characteristics of the participants without MMSE data at the first follow-up are shown in Supplementary Table 2.

**Table 1.** Baseline Characteristics of the Participants Per Cohort

	LASA (*N* = 1,736)	InCHIANTI (*N* = 843)	Rotterdam Study (*N* = 4,597)
Age at baseline (years), Mean (*SD*)	69.8 (8.1)	72.4 (7.2)	67.8 (7.4)
Female, *N* (%)	896 (51.6%)	470 (55.7%)	2,617 (56.9%)
*APOE* ɛ _4_ carriers, *N* (%)	467 (26.9%)	128 (15.2%)	1,268 (27.6%)
Physical activity	Range (MET h/wk)	*N* (%)	Range (MET h/wk)
Low	0.2–41.4	122 (14.5%)	2.8–63.4
Moderate	41.5–74.8	383 (45.4%)	63.5–99.3
High	74.9–325.5	338 (40.1%)	99.4–296.3
MMSE at baseline, Mean (*SD*)	27.5 (2.1)	25.9 (2.7)	27.9 (1.7)
Education, *N* (%)			
Low (<9 y)	685 (39.5%)	742 (88.0%)	505 (11.1%)
Intermediate (9–12 y)	835 (48.2%)	43 (5.1%)	2,025 (44.4%)
High (>12 y)	214 (12.3%)	58 (6.9%)	2,030 (44.5%)
Depression, *N* (%)	213 (12.3%)	245 (29.1%)	428 (9.4%)
Number of chronic disease, *N* (%)			
0–1	1,343 (77.4%)	545 (69.3%)	4,080 (88.8%)
2	279 (16.1%)	147 (18.7%)	428 (9.3%)
>2	113 (6.5%)	95 (12.0%)	89 (1.9%)

*Note*: Low physical activity(PA) corresponds to the inactive category for InCHIANTI and first tertile for LASA and RS; Moderate PA corresponds to the light PA category for InCHIANTI and to the second tertile for LASA and RS; High PA corresponds to the moderate–high category for InCHIANTI and the highest tertile in LASA and RS.

Mean age at baseline was higher for InCHIANTI participants (72.4 [7.2] years) compared to LASA (69.8 [8.1] years) and RS (67.8 [7.4] years). MMSE score at baseline was lower (25.9 [2.7]) for InCHIANTI compared to LASA (27.5 [2.1]) and RS participants (27.9 [1.7]). Most InCHIANTI participants (88%) had completed up to 9 years of education while most LASA and RS participants had attained more than 9 years of education. LASA and RS participants had a similar percentage of *APOE* ɛ _4_ carriers, respectively 26.9% and 27.6%, while only 15.2% of the respondents were carriers in InCHIANTI (Table 1). Participants who did not have MMSE data at the first follow-up were on average older, more often *APOE* ɛ _4_ carriers, and had more often depression or chronic diseases (Supplementary Table 2).

**Figure 1.**
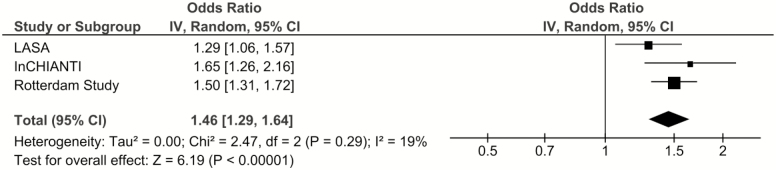
The association between *APOE* ɛ _4_ and cognitive decline: Results from three longitudinal cohort studies. Model 2: Results adjusted for age, sex, education, depression, and chronic diseases.

The unadjusted effect estimates are presented in Supplementary Table 3. In the pooled adjusted analysis, *APOE* ɛ _4_ carriers had higher odds of cognitive decline (odds ratio [OR] = 1.46, 95% confidence interval [CI]: 1.29, 1.64) compared to noncarriers and the results were similar across cohorts (I^2^ = 19%, *p* = .29; Figure 1).

**Figure 2.**
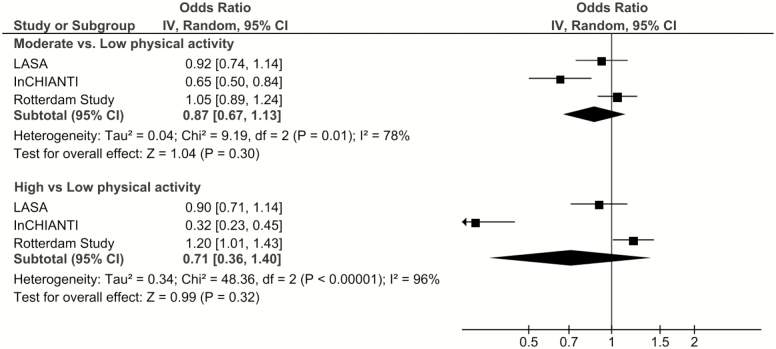
The association between physical activity and cognitive decline: Results from three longitudinal cohort studies. Model 2: Results adjusted for age, sex, education, depression, and chronic diseases.

Overall, there was no association between PA and cognitive decline in the pooled analysis (moderate PA: OR = 0.87, 95% CI: 0.67–1.13; high PA: OR = 0.71, 95% CI: 0.36–1.40), but the heterogeneity between studies was substantial (moderate PA: I^2^ = 78%, *p* = .01; high PA: I^2^ = 96%, *p* < .0001; Figure 2). Similarly, there was no association between PA and cognitive decline when only LASA and InCHIANTI, LASA, and RS or InCHIANTI and RS were meta-analyzed (leave-one-out meta-analysis, Supplementary Table 4). In InCHIANTI, higher PA was associated with lower odds of cognitive decline (high PA compared to low PA, OR = 0.32, 95% CI: 0.23, 0.44), whereas no such associations were seen in LASA and RS.

**Figure 3.**
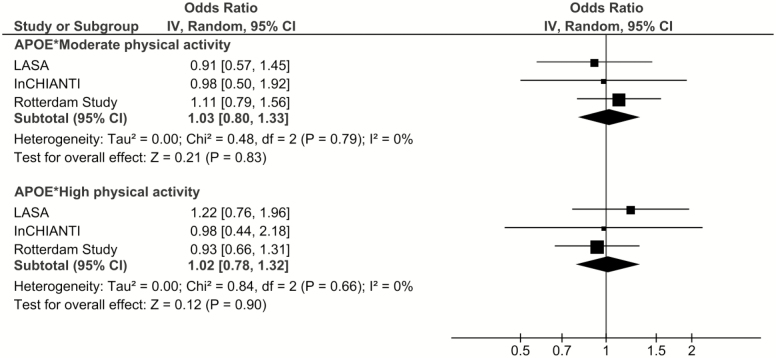
The association between the interaction effect of *APOE* and physical activity and cognitive decline: Results from three longitudinal studies. Model 2: Results adjusted for age, sex, education, depression, and chronic diseases.

No gene-by-environment interaction between *APOE* and PA in cognitive decline was found in multiplicative scale (*APOE* × moderate PA: OR = 1.03, 95% CI: 0.80–1.33; *APOE* × high PA: OR = 1.02, 95% CI: 0.78–1.32) and there was no heterogeneity between studies (*APOE* × moderate PA: I^2^ = 0.0%, *p* = .79; *APOE* × high PA: I^2^ = 0.0%, *p* = .66; Figure 3).

Moreover, no evidence for interaction on additive scale between *APOE* and PA was found in LASA or InCHIANTI (LASA: moderate PA: RERI = −0.11, 95% CI: −0.75, 0.42; high PA: RERI = 0.20, 95% CI: −0.37, 0.59; InCHIANTI: moderate PA: RERI = −0.28, 95% CI: −1.70, 0.53; high PA: RERI = −0.50, 95% CI: −1.92, 0.28).

### Sensitivity Analysis

Analyses including only cognitively healthy individuals at baseline (MMSE ≥24) showed similar results (Supplementary Table 5).

Furthermore, also the association of PA and *APOE ×* PA assessed using PA in continuous scale in LASA and RS was consistent with the main findings (Supplementary Table 6).

### Extra Analysis in LASA

In line with the main analyses, *APOE* ɛ _4_ was associated with more decline in total recall score, delayed recall score, and information processing speed. There was no association of PA with specific aspects of cognition and no GxE interaction between *APOE* and PA in total recall score, delayed recall score or information processing speed was found (Supplementary Table 7).

## Discussion

In this large study conducted among older adults in three prospective population-based cohort studies, we aimed to explore whether PA might moderate the effect of *APOE* genotype on cognitive decline. The present findings confirmed that *APOE* ɛ _4_ allele was associated with higher risk of cognitive decline. Nevertheless, the GxE interaction between *APOE* genotype and PA was not associated with cognitive decline. Thus, the hypothesis that PA might modify the association between APOE and cognitive decline was not supported by our results.

Our results are in line with the findings from Whitehall II study (40), which reported no interaction between *APOE* ɛ _4_ and PA in cognitive function in late midlife as well as with the findings from UK Biobank (41), which reported no interaction between *APOE* ɛ _4_ and PA in cognitive abilities in persons aged 40–70 years. However, compared to our study, both studies had younger participants and did not have longitudinal data on cognitive function. Luck and colleagues analyzed data from the German study on Ageing, Cognition and Dementia in Primary care Patients (AgeCoDe) and reported no GxE interaction in multiplicative scale between *APOE* ɛ _4_ and PA in dementia but they found a possible additive interaction in Alzheimer’s disease (15). In contrast to our findings, a cohort study of 347 older Dutch men found a stronger protective effect of PA in cognitive decline in *APOE* ɛ _4_ carriers compared to noncarriers (31). The participants in this study were on average older, the follow-up period was 3 years compared to 9 years in our study and cognitive decline was assessed only once. The different outcome definitions used in the literature, the age of the participants, the follow-up time and physical activity assessment make it difficult to directly compare the results. To date, it can be postulated that the GxE interaction is present in AD but there is no evidence of this interaction in cognitive decline and cognitive functioning.

Overall, we found no association between PA and cognitive decline; however, the results were heterogeneous between the participating cohorts. PA was strongly associated with lower odds of cognitive decline in InCHIANTI while in LASA and RS this association was not statistically significant. The differences may be attributed to different questionnaires used per study or to specific population characteristics (ie, older age, lower educational level), suggesting that study characteristics should be taken into account when comparing results across studies.

Even though the majority of studies in the literature support a protective effect of PA in dementia and cognitive decline, the number of observational studies reporting no effect has increased in recent years. The evidence for a short-term effect (<5 years) is quite robust but there seems to be no effect in studies using a longer follow-up. In a recent publication from the Whitehall II study PA did not decrease the risk of dementia after 28 years follow-up (42). Likewise, when examining the association between PA and risk of dementia in RS, there was a short-term protective effect of PA using a follow-up up to 4 years but no association for longer follow-up (29). Furthermore, evidence from clinical trials does not support a long-term protective effect of PA on dementia or cognitive decline for follow-up 1 year or longer (43).

Strengths of our study include its prospective design, long follow-up period, large sample size, and large number of observations. To the best of our knowledge, this is the first study to analyze the GxE interaction between PA and *APOE* ε _4_ in cognitive decline in three similar European, population-based, cohort studies. Variations in measurement methods were taken into account in the analysis plan by creating similar exposure, outcome, and covariate variables per study.

Nevertheless, the current study has some limitations. First, PA was assessed with self-reported questionnaires, which can be prone to information bias. Correlation of LAPAQ questionnaire with pedometer data in LASA was 0.56 (24), whereas the correlation of LAPAQ with wrist accelerometer data in RS was 0.3 (44). Second, each study assessed PA with different questionnaires, which may have contributed to heterogeneity in results. This was taken into account by using random effects meta-analysis to pool the data. Third, the MMSE is designed as a cognitive screening tool, and may not be very sensitive to assess decline in cognitive function. However, extra analysis using more specific tests such as 15 Word Test and Alphabet Coding Task-15 performed in LASA show similar results. Fourth, reverse causality might be present in the association between PA and cognitive decline. To account for possible reverse causation, a sensitivity analysis including only cognitively healthy participants (baseline MMSE ≥24) was performed and the results were similar with the main analysis. Fifth, our cohorts included only European ancestry participants and generalizability of the results in non-European populations should be taken with caution.

Assessment of PA with more objective measurements is needed in the future as these may better reflect the total amount of PA. Moreover, using a more sensitive test to assess cognitive decline can minimize the misclassification of the outcome and detect associations with more precision.

In spite of previous evidence suggesting a beneficial effect of PA in *APOE* ɛ _4_ carriers, we found no evidence for a moderation effect by PA in the association between *APOE* genotype and cognitive decline. Based on our pooled results, PA is not associated with cognitive decline in older adults even though there was heterogeneity across the three cohorts. Further research is needed to identify subgroups where PA can protect against cognitive decline or type of activities that are more beneficial for preventing cognitive decline. Meanwhile PA in older adults should be encouraged for its protective effects in other conditions present at this age group.

In conclusion, the claims of a gene–environment interaction between *APOE* ɛ _4_ and PA in cognitive decline are not supported by our results. There is a need for large sample replications before translating results from candidate GxE studies into clinical recommendations.

## Supplementary Material

glaa054_suppl_Supplementary_material
