# Supplementary material for: Physical Activity as Moderator of the Association Between APOE and Cognitive Decline in Older Adults: Results from Three Longitudinal Cohort Studies
Source: J Gerontol A Biol Sci Med Sci. 2020 Feb 28;75(10):1880–6. doi: 10.1093/gerona/glaa054 (PMC7518558; doi:10.1093/gerona/glaa054)
Supplement: glaa054_suppl_Supplementary_material [file glaa054_suppl_supplementary_material.docx]

Supplementary Material

SUPPLEMENTARY METHODS

Study description

Longitudinal Aging Study of Amsterdam (LASA) is an ongoing, population-based, cohort study of individuals 55 years and older living in Amsterdam, Zwolle and Oss in the Netherlands. The design and rationale are described by Hoogendijk et al. (1). In short, 3017 participants were included at baseline (1992) and follow-up visits were conducted every 3 years. Trained interviewers collected data on cognitive, emotional, physical and social functioning during a home interview. Subsequently, all participants were invited for a medical interview during which further diagnostic examinations were done and blood samples were drawn.

LASA has been approved by the Medical Ethics Committee of VU University Medical Center and all participants gave written informed consent.

Invecchiare in Chianti (InCHIANTI) is an ongoing, population-based, cohort study of individuals selected from the population of Greve in Chianti and Bagno a Ripoli, Tuscany, Italy. The design and rationale are described in details elsewhere (2). In brief, 1453 participants were included in the period September 1998-March 2000. Interviews were conducted at the participants’ homes by three experienced interviewers and those who scored 18 or less at the Mini-Mental State Examination were interviewed with a proxy. The home interviews were conducted to collect information on architectural barriers, household composition and social networks, depressive symptoms, ability to perform daily life activities, foot problems, falls and fear of falling, health-related behaviors, pharmacological treatments, incontinence, quality of sleep and food intake. Within the following three weeks after interview, participants underwent a detailed clinical examination and several blood samples were drown. Follow-up visits were held every 3 years.

InCHIANTI study protocol has been approved by the Italian National Research Council on Aging Ethical Committee and it is in line with the Declaration of Helsinki for human research. All participants gave written informed consent at each visit to participate in the study, permission to consult past and future medical files and to conduct analysis in blood and urine samples, including DNA analysis.

Rotterdam Study (RS) is an ongoing, population-based, cohort study of individuals 55 years and older living in Ommoord district of Rotterdam, the Netherlands. The design and rationale are described in details elsewhere (3). In brief, an initial cohort of 7983 participants was established between 1990-1993. Trained research assistants collected data on medical history, current health status, use of medication and lifestyle factors during the home interview. Afterwards participants were invited to the study center where they underwent a detailed clinical examination and blood samples were drawn. Follow-up visits were held every 4 years.

The Rotterdam Study has been approved by the Medical Ethics Committee according to the Wet Bevolkingsonderzoek: ERGO (Population Study Act: Rotterdam Study), executed by the Ministry of Health, Welfare and Sports of the Netherlands. All participants gave informed consent to participate in the study and to obtain information from treating physicians and pharmacies.

Assessment of covariates

LASA: To assess educational attainment participants were asked how many years of education had they completed (years, continuous). Depressive symptoms were assessed at baseline using the Center for Epidemiologic Studies Depression scale (CES-D)(4). A score of 16 or higher was considered as clinically relevant depressive symptoms. Presence of chronic disease was assessed at the main interview by asking participants if they had any of the following chronic disease: chronic non-specific pulmonary disease, asthma, cardiac disease, cerebrovascular accident or stroke, peripheral arterial disease, diabetes mellitus, cancer, osteoarthritis or rheumatoid arthritis.

InCHIANTI: As covariates of decline of MMSE, we evaluated at baseline: socio-demographic variables (age, gender and education (recorded in years)), number of diseases based on medical conditions self-reported history, clinical documentation and medication use (the following were considered: Cancer, Angina pectoris, Myocardial infarction, Congestive heart failure, Stroke, Peripheral arterial disease (PAD), Diabetes mellitus Type 2, Chronic bronchitis or emphysema, Bronchial asthma and Possible osteoporosis detected via pQCT), and depressive symptoms assessed by the CES-D.

RS: Educational attainment was ascertained at study entry by interview and classified into four categories corresponding to 7 years (i.e. primary education only), 10 years (lower or intermediate general education or lower vocational education), 13 years (intermediate vocational education or higher general education), and 19 years (higher vocational or university education) of education, respectively. Depressive symptoms were assessed at baseline using the CES-D. Chronic obstructive pulmonary disease was diagnosed based on an obstructive pre-bronchodilator spirometry (FEV1/FVC<0.70) according to the GOLD guidelines(5). Diagnosis of heart disease was based on self-reported history, electrocardiography, and inspection of medical records(6). Peripheral arterial disease was based on self-reported history requiring surgery or stent placement. Diabetes was defined as the use of blood glucose-lowering medication, a fasting serum glucose level ≥7.0 mmol/L (126 mg/dL), or a non-fasting serum glucose level ≥11.1 mmol/L (200 mg/dL). A history of cancer was assessed by interview, inspection of medical records, and linkage to the Dutch cancer registry. Stroke was diagnosed on the basis of self-reported diagnoses, and inspection of medical records. A diagnosis of osteoarthritis in knee or hip required radiographic evidence and pain in the affected joint.Supplementary Figure 1: Flow chart of participants included in the analysis

Participants ≥55 years included at baseline:

N_LASA_= 3107

N_InCHIANTIi_= 1235

N_RS_= 7510

No *APOE* data available:

N_LASA_= 874

N_InCHIANTI_= 111

N_RS_= 643

Included participants with *APOE* data available:

N_LASA_= 2233

N_InCHIANTI_= 1124

N_RS_= 6867

No baseline MMSE data available or baseline MMSE <18:

N_LASA_= 37

N_InCHIANTI_= 84

N_RS_= 578

Included participants with *APOE* data available and baseline MMSE ≥18

N_LASA_= 2196

N_InCHIANTI_= 1040

N_RS_= 6289

No baseline physical activity data or are bedridden or in a wheelchair:

N_LASA_=117

N_InCHIANTI_= 39

N_RS_= 97

Included participants with *APOE*, MMSE and physical activity data available:

N_LASA_= 2079

N_InCHIANTI_= 1001

N_RS_= 6192

Non-Caucasian ethnicity:

N_LASA_= 16

N_InCHIANTI_= 0

N_RS_= 252

Included participants of Caucasian ethnicity and *APOE*, MMSE and physical activity available:

N_LASA_= 2063

N_InCHIANTI_= 1001

N_RS_= 5940

No MMSE data at the first follow-up visit:

N_LASA_= 327

N_InCHIANTI_= 158

N_RS_= 1343

Included participants in the main analysis:

N_LASA_= 1736

N_InCHIANTI_= 843

N_RS_= 4597

Supplementary Table 1: Overview of methods and instruments used to assess exposure, outcome and covariate data in LASA, InCHIANTI and RS.

|  | **LASA** | **InCHIANTI** | **RS** |
| --- | --- | --- | --- |
| Number of participants | 1,736 | 843 | 4,597 |
| Total number of observation | 4,255 | 2,132 | 12,102 |
| Baseline measurement | 1992-1993 | 1998- 2000 | 1997-2001 |
| Follow-up 1 | 1995-1996 | 2001-2003 | 2002-2005 |
| Follow-up 2 | 1998-1999 | 2004-2006 | 2008-2014 |
| Follow-up 3 | 2001-2002 | 2007-2010 | - |
| Number of participants at baseline | 2063 | 1001 | 5940 |
| Number of participants at follow-up 1 | 1736 | 843 | 4597 |
| Number of participants at follow-up 2 | 1442 | 705 | 3007 |
| Number of participants at follow-up 3 | 1148 | 603 | - |
| Total follow-up period (years) | 9 years | 9 years | ~ 10 years |
| *APOE* status | Phenotyping confirmed by genotyping using Axiom-NL array by Affymetrix or Global Screening Array by Illumina. Discordance genotyping-phenotyping in 1021 individuals was 3.1%. | Genotyping, using Illumina Infinium HumanHap550 chip. | Single gene genotyping, using polymerase chain reaction. |
| Physical activity (PA) | LASA physical activity questionnaire (LAPAQ), categorized in tertiles based on the total METhours/week. | Modified EPIC-Norfolk physical activity questionnaire, categorized in  a) inactive (mostly sitting/some walking)  b) light physical activity  c) moderate-high physical activity | Zutphen physical activity questionnaire,  categorized in tertiles based on the total METhours/week. |
| Cognitive decline | Cognitive function assessed with MMSE. A decrease of 3 or more points in MMSE in the following visit is considered as cognitive decline. | Cognitive function assessed with MMSE. A decrease of 3 or more points in MMSE in the following visit is considered as cognitive decline. | Cognitive function assessed with MMSE. A decrease of 3 or more points in MMSE in the following visit is considered as cognitive decline. |
| Education | Assessed in years of education completed. Categorized in:   - - Low : <9 years of education completed   - Intermediate: 9-12 years of education completed   - High : > 12 years of education completed | Assessed in years of education completed. Categorized in:   - - Low : <9 years of education completed   - Intermediate: 9-12 years of education completed   - High : > 12 years of education completed | Assessed in categories: primary education only (7 years), lower or intermediate general education or lower vocational education (10 years), intermediate vocational or higher general education (13 years), higher vocational education or university (19 years).Subsequently, the two last categories were merged into one. |
| Chronic disease  The following disease were assessed: Chronic obstructive pulmonary disease, asthma, chronic bronchitis or emphysema, angina pectoris, myocardial infarction, congestive heart failure, stroke, peripheral arterial disease, type 2 diabetes, cancer, osteoarthritis or rheumatoid arthritis (and possible osteoporosis for InCHIANTI). | Self –reported , categorized in :   - 0-1 chronic disease   - 2 chronic disease   - >2 chronic disease | A combination of self-reported, clinical documentation and medication use data,  categorized in :   - 0-1 chronic disease   - 2 chronic disease   - >2 chronic disease | A combination of self-reported, clinical documentation, specific tests and medication use data,  categorized in :   - 0-1 chronic disease   - 2 chronic disease   - >2 chronic disease |
| Clinically relevant depressive symptoms | CES-D , a score ≥16 is considered as clinically relevant. | CES-D , a score ≥16 is considered as clinically relevant. | CES-D , a score ≥16 is considered as clinically relevant. |

CES-D: Center for Epidemiologic Studies- Depression Scale; MMSE: Mini-Mental State Examination.

Supplementary Table 2: Baseline characteristics of the participants with no MMSE data at the first follow-up.

|  | **LASA**  **(N=327)** | **InCHIANTI**  **(N=158)** | **Rotterdam Study**  **(N=1343)** |
| --- | --- | --- | --- |
| Age at baseline (years), Mean(SD) | 73.6 (8.1) | 75.7 (8.4) | 74.2 (8.8) |
| Female, N(%) | 144 (44%) | 87 (55.1%) | 726 (54.1%) |
| *APOE* ɛ_4_ carriers, N(%) | 89 (27.2%) | 26 (16.5%) | 399 (29.7%) |
| Physical activity, MET/hours week Mean (SD) | 61.0 (44.0) | N (%) | 72.7 (44.5) |
| Low |  | 36 (22.8%) |  |
| Moderate |  | 64 (40.5%) |  |
| High |  | 58 (36.7%) |  |
| MMSE at baseline,  Mean, (SD) | 26.6 (2.4) | - | 27 (2.0) |
| Education, years completed, Mean (SD) | 8.3 (3.1) | 5.41 (3.42) | 11.2 (3.4) |
| Depression, N(%) | 45 (13.8%) | 49 (31.2%) | 175 (13.2%) |
| Nr of chronic disease, N(%) |  |  |  |
| 0-1 | 227 (69.4%) | 75 (55.6%) | 1086 (80.9%) |
| 2 | 65 (19.9%) | 27 (20%) | 193 (14.4%) |
| >2 | 34 (10.4%) | 33 (24.4%) | 64 (4.8%) |

Note: Physical activity was assessed in categories for InCHIANTI and in MET/hours per week for LASA and Rotterdam study.

Supplementary Table 3: The association between *APOE*, physical activity and *APOE**physical activity interaction; Unadjusted results from 3 longitudinal cohort studies.

| Unadjusted results: OR (95%CI)  APOE | |
| --- | --- |
| LASA | 1.173 (0.964, 1.427) |
| InCHIANTI | 1.501 (1.146, 1.965) |
| Rotterdam Study | 1.442 (1.255, 1.658) |
| Pooled | 1.360 (1.177, 1.572) |
| Moderate physical activity, OR (95%CI) | |
| LASA | 0.850 (0.690, 1.048) |
| InCHIANTI | 0.531 (0.417, 0.675) |
| Rotterdam Study | 0.959 (0.815, 1.130) |
| Pooled | 0.764 (0.548, 1.063) |
| High physical activity | |
| LASA | 0.698 (0.562, 0.865) |
| InCHIANTI | 0.241 (0.178, 0.328) |
| Rotterdam Study | 1.032 (0.879, 1.212) |
| Pooled | 0.564 (0.267, 1.188) |
| APOE*Moderate physical activity | |
| LASA | 0.973 (0.604, 1.567) |
| InCHIANTI | 1.397 (0.507, 1.879) |
| Rotterdam Study | 1.113 (0.789, 1.568) |
| Pooled | 1.084 (0.828, 1.418) |
| APOE*High physical activity | |
| LASA | 1.422 (0.886, 2.282) |
| InCHIANTI | 1.498 (0.467, 2.278) |
| Rotterdam Study | 0.921 (0.653, 1.298) |
| Pooled | 1.114 (0.810, 1.534) |

Supplementary Table 4: The main effect of physical activity on cognitive decline: Leave-one-out meta-analysis results.

| **Cohorts** | **Moderate physical activity** | **High physical activity** |
| --- | --- | --- |
| **LASA and InCHIANTI** | | |
| OR (95%CI) | 0.78 (0.55,1.10) | 0.54 (0.20,1.49) |
| I^2^, (*P-value*) | 75% (0.04) | 96% (<0.001) |
| **LASA and Rotterdam Study** | | |
| OR (95%CI) | 1.00 (0.88,1.14) | 1.05(0.79,1.39) |
| I^2^, (*P-value*) | 0% (0.34) | 73% (0.05) |
| **InCHIANTI and Rotterdam Study** | | |
| OR (95%CI) | 0.84 (0.52,1.34) | 0.62 (0.17,2.28) |
| I^2^, (*P-value*) | 89% (0.002) | 98% (<0.001) |

Adjusted for age, sex, education, depression and chronic diseases.

Supplementary Table 5: Sensitivity analysis: The association between *APOE*, PA and *APOE**PA interaction in individuals with baseline MMSE ≥24; Results from 3 longitudinal cohort studies.

|  | **Model 1**  **OR (95% CI)** | **Model 2**  **OR (95% CI)** |
| --- | --- | --- |
| ***APOE*** |  |  |
| *LASA (N=1640)* | 1.337 (1.093, 1.636) | 1.328 (1.087, 1.624) |
| *InCHIANTI (N=682)* | 1.634 (1.193, 2.237) | 1.640 (1.197, 2.246) |
| *Rotterdam Study (N=4494)* | 1.505 (1.307, 1.734) | 1.517 (1.318, 1.749) |
| *Pooled* | 1.469 (1.318, 1.637) | 1.473 (1.322, 1.641) |
| **Physical activity** |  |  |
| *LASA* |  |  |
| Moderate | 0.896 (0.721, 1.113) | 0.881 (0.706, 1.098) |
| High | 0.859 (0.681, 1.085) | 0.842 (0.664, 1.067) |
| *InCHIANTI* |  |  |
| Moderate | 0.612 (0.457, 0.821) | 0.618 (0.461, 0.830) |
| High | 0.308 (0.212, 0.446) | 0.310 (0.213, 0.452) |
| *Rotterdam Study* |  |  |
| Moderate | 1.013 (0.857, 1.198) | 1.023 (0.865, 1.211) |
| High | 1.158 (0.979, 1.370) | 1.165 (0.984, 1.380) |
| *Pooled* |  |  |
| Moderate | 0.841 (0.645, 1.096) | 0.842 (0.644, 1.099) |
| High | 0.689 (0.366, 1.296) | 0.687 (0.363, 1.301) |
| ***APOE**PA interaction** |  |  |
| *LASA* |  |  |
| *APOE**Moderate PA | 0.816 (0.500, 1.333) | 0.810 (0.497, 1.322) |
| *APOE**High PA | 1.126 (0.696, 1.823) | 1.106 (0.683, 1.791) |
| *InCHIANTI* |  |  |
| *APOE**Moderate PA | 1.167 (0.530, 2.570) | 1.153 (0.524, 2.541) |
| *APOE**High PA | 1.274 (0.499, 3.116) | 1.233 (0.493, 3.085) |
| *Rotterdam Study* |  |  |
| *APOE**Moderate PA | 1.125 (0.794, 1.595) | 1.132 (0.799, 1.606) |
| *APOE**High PA | 0.936 (0.661, 1.326) | 0.940 (0.663, 1.331) |
| *Pooled* |  |  |
| *APOE**Moderate PA | 1.027 (0.786, 1.341) | 1.027 (0.786, 1.341) |
| *APOE**High PA | 1.018 (0.777, 1.333) | 1.013 (0.773, 1.327) |

Model 1: Adjusted for age, sex and education

Model 2: Model 1 + additionally adjusted for depression and number of chronic disease

Supplementary table 6: The association between continuous PA, *APOE**PA and cognitive decline; Results from LASA and Rotterdam Study

|  | **Model 1**  **OR (95% CI)** | **Model 2**  **OR (95% CI)** |
| --- | --- | --- |
| **Physical activity (PA/SD)** |  |  |
| LASA | 1.02 (0.922, 1.116) | 1.01 (0.920, 1.116) |
| Rotterdam Study | 1.07 (0.999, 1.145) | 1.07 (1.001, 1.148) |
| Pooled | 1.05 (0.996, 1.115) | 1.05 (0.994, 1.108) |
| *I^2^ (p-value)* | 0.0% (0.44) | 0.0% (0.32) |
| ***APOE** physical activity** |  |  |
| LASA | 1.14 (0.941, 1.380) | 1.14 (0.938, 1.375) |
| Rotterdam Study | 0.99 (0.857, 1.135) | 0.99 (0.856, 1.136) |
| Pooled | 1.04 (0.910, 1.199) | 1.04 (0.910, 1.198) |
| *I^2^ (p-value)* | 28.9% (0.24) | 26.9% (0.24) |

PA; physical activity, SD; standard deviation

Model 1: Adjusted for age, sex and education

Model 2: Model 1 + additionally adjusted for depression and number of chronic disease

Supplementary table 7: The association between *APOE*, PA and *APOE*PA* interaction with memory and information processing speed in LASA participants.

|  | **Model 1**  **B (95% CI)** | **Model 2**  **B (95% CI)** |
| --- | --- | --- |
| **Change in recall score** |  |  |
| *APOE* ɛ_4_ | 0.397 (0.098,0.696) | 0.403 (0.104,0.702) |
| Physical activity  (PA/SD) | 0.057 (-0.083,0.198) | 0.049 (-0.092,0.190) |
| *APOE** physical activity | -0.015 (-0.316,0.286) | 0.005 (-0.297, 0.306) |
| **Change in delayed recall score** |  |  |
| *APOE* ɛ_4_ | 0.156 (0.005,0.306) | 0.159 (0.008,0.310) |
| Physical activity  (PA/SD) | 0.008 (-0.063,0.079) | 0.008 (-0.064,0.079) |
| *APOE** physical activity | -0.008 (-0.169,0.154) | -0.004 (-0.166,0.158) |
| **Change in information processing speed** |  |  |
| *APOE* ɛ_4_ | 0.371 (0.155,0.587) | 0.381 (0.162,0.599) |
| Physical activity  (PA/SD) | -0.045 (-0.135,0.046) | -0.038 (-0.129,0.052) |
| *APOE** physical activity | 0.030 (-0.174,0.234) | 0.036 (-0.167, 0.240) |

PA; physical activity, SD; standard deviation

Model 1: Adjusted for age, sex and education

Model 2: Model 1 + additionally adjusted for depression and number of chronic disease

REFERENCES

1. Hoogendijk EO, Deeg DJ, Poppelaars J, van der Horst M, Broese van Groenou MI, Comijs HC, et al. The Longitudinal Aging Study Amsterdam: cohort update 2016 and major findings. Eur J Epidemiol. 2016;31(9):927-45.

2. Ferrucci L, Bandinelli S, Benvenuti E, Di Iorio A, Macchi C, Harris TB, et al. Subsystems contributing to the decline in ability to walk: bridging the gap between epidemiology and geriatric practice in the InCHIANTI study. J Am Geriatr Soc. 2000;48(12):1618-25.

3. Ikram MA, Brusselle GGO, Murad SD, van Duijn CM, Franco OH, Goedegebure A, et al. The Rotterdam Study: 2018 update on objectives, design and main results. Eur J Epidemiol. 2017;32(9):807-50.

4. Radloff LS. The CES-D Scale. Applied Psychological Measurement. 1977;1(3):385-401.

5. Terzikhan N, Verhamme KM, Hofman A, Stricker BH, Brusselle GG, Lahousse L. Prevalence and incidence of COPD in smokers and non-smokers: the Rotterdam Study. Eur J Epidemiol. 2016;31(8):785-92.

6. Leening MJ, Kavousi M, Heeringa J, van Rooij FJ, Verkroost-van Heemst J, Deckers JW, et al. Methods of data collection and definitions of cardiac outcomes in the Rotterdam Study. Eur J Epidemiol. 2012;27(3):173-85.
